# Coulombic self-ordering upon charging a large-capacity layered cathode material for rechargeable batteries

**DOI:** 10.1038/s41467-019-09409-1

**Published:** 2019-05-16

**Authors:** Benoit Mortemard de Boisse, Marine Reynaud, Jiangtao Ma, Jun Kikkawa, Shin-ichi Nishimura, Montse Casas-Cabanas, Claude Delmas, Masashi Okubo, Atsuo Yamada

**Affiliations:** 1 Department of Chemical System Engineering, School of Engineering, The University of Tokyo, Hongo 7-3-1, Bunkyo-ku, Tokyo 113-8656 Japan; 2 CIC energiGUNE, Parque Tecnológico de Álava, 01510 Vitoria-Gasteiz, Álava Spain; 3 Advanced Key Technologies Division, National Institute for Materials Science, Tsukuba, Ibaraki 305-0044 Japan; 4 Elements Strategy Initiative for Catalysts & Batteries (ESICB), Kyoto University, Nishikyo-ku, Kyoto 614-8245 Japan; 5 Institut de Chimie de la Matière Condensée de Bordeaux (ICMCB), 33600 Pessac, France

**Keywords:** Materials science, Materials for energy and catalysis, Batteries

## Abstract

Lithium- and sodium-rich layered transition-metal oxides have recently been attracting significant interest because of their large capacity achieved by additional oxygen-redox reactions. However, layered transition-metal oxides exhibit structural degradation such as cation migration, layer exfoliation or cracks upon deep charge, which is a major obstacle to achieve higher energy-density batteries. Here we demonstrate a self-repairing phenomenon of stacking faults upon desodiation from an oxygen-redox layered oxide Na_2_RuO_3_, realizing much better reversibility of the electrode reaction. The phase transformations upon charging A_2_MO_3_ (A: alkali metal) can be dominated by three-dimensional Coulombic attractive interactions driven by the existence of ordered alkali-metal vacancies, leading to counterintuitive self-repairing of stacking faults and progressive ordering upon charging. The cooperatively ordered vacancy in lithium-/sodium-rich layered transition-metal oxides is shown to play an essential role, not only in generating the electro-active nonbonding 2*p* orbital of neighbouring oxygen but also in stabilizing the phase transformation for highly reversible oxygen-redox reactions.

## Abstract (web summary)

Here the authors report the evolution of stacking faults in Na_2_RuO_3_ showing that there is progressive cation ordering upon charging and notably the stacking faults can disappear. This behavior is driven by cooperative Coulombic interactions and can contribute to stabilizing the phase transformations.

## Introduction

The discovery of intercalation chemistry in layered transition-metal oxides AMO_2_ (A=Li, Na and M=transition metal)^1,2^ in the early 1980s has led to the commercialization of lithium-ion batteries^3^. Tremendous effort has since been devoted to understanding how alkali-metal ions reversibly (de)intercalate in AMO_2_ because this is essential to exploit their large theoretical capacities (~275 mAh g^−1^ for LiCoO_2_ and ~235 mAh g^−1^ for NaCoO_2_, respectively). It is now well understood that, at the early stage of A^+^ deintercalation, A_*x*_MO_2_ (0.4 < *x* < 1.0) exhibit an increase of their interlayer distance because the depletion of screening A^+^ layers enhances the effective Coulombic repulsion between oxide ions of adjacent MO_2_ layers^4–8^. At the late stage of *A*^+^ deintercalation (0.0 < *x* < 0.4), high-valent M increases the covalency of M*−*O bonds, and thus decreases the negative charge on oxide ions. In this situation, O*−*O van der Waals attraction forces are not sufficient to maintain the layered structure, and the large volume variations induced at the end of charge often initiate crack formation and delamination/exfoliation^8–10^. Furthermore, the lack of alkali ions in the interlayer space leads to structural degradation with migration of transition-metal ions to neighboring tetrahedral sites^11–13^. This established knowledge on the intercalation chemistry of A_*x*_MO_2_ explains the practical limit of their reversible capacity (approximately for 0.4 < *x* < 1.0, i.e., 170 mAh g^−1^ for A = Li and 140 mAh g^−1^ for A = Na). Therefore, the control of the competing Coulombic and van der Waals forces in layered transition-metal oxides is of great importance to achieve a large reversible capacity.

Layered *A*-excess transition-metal oxides (A_1+*y*_M_1−*y*_O_2_ or A_1_[A_*y*_M_1−*y*_]O_2_) are recent major targets to increase the cathode capacity by virtue of additional oxygen-redox reactions. Li_2_MnO_3_-LiMO_2_ solid solutions have been reported to deliver large capacities over 200 mAh g^−1^^14–16^, and have more recently been followed by Li_2_MO_3_ (M = Ru^17^, Ir^18^, RuSn^17^, and RuMn^19^) and Na_2_MO_3_ (M = Ru^20,21^, RuSn^21^, and Ir^22^), all delivering large capacities exceeding that of solely M redox. Although the changes in the electronic state during the additional oxygen-redox reactions have been intensively investigated, less attention has been paid to the essential interaction dominating the phase transformation during the charge/discharge processes, presumably because most oxygen-redox electrodes exhibit severe structural degradation (i.e., oxygen-gas evolution, cation migration) at the initial charge^16,17,23,24^. However, the extra A^+^ are expected to play a crucial role in the structural transformation during the charge/discharge processes. For example, the depletion of screening A^+^ layers upon charging can be compensated by the A^+^ supplied  from the [A_*y*_M_1−*y*_]O_2_ layers^18,20,22^. Moreover, A^+^ (or vacancy after deintercalation) in the [A_*y*_M_1−*y*_]O_2_ layers is expected to modulate the balance of competing Coulombic/van der Waals forces, and hence largely influence the intercalation chemistry.

We have recently studied the structure and electrochemistry of O3-Na_2_RuO_3_ (or Na[Na_1/3_Ru_2/3_]O_2_), where [Na_1/3_Ru_2/3_]O_2_ layers have a honeycomb-type ordered arrangement of Na and Ru^20,25^. According to the classification of layered oxides, O3 denotes a structure where Na^+^ ions occupy octahedral interlayer sites and the stacking of oxide ions is ABCABC (Supplementary Fig. 1)^26^. Importantly, in contrast to most oxygen-redox electrodes, O3-Na_2_RuO_3_ exhibits highly reversible (de)sodiation without structural degradation but rather exhibits progressive structural ordering upon charging, which provides an opportunity for not only detailed structural investigation as a model system but also for essential strategies toward much larger reversible capacity.

In this work, synchrotron X-ray diffraction coupled with planar-defect refinement analyses are applied to honeycomb ordered Na_*x*_RuO_3_ phases (*x* = 2, 1, and 1/2), revealing a self-repairing phenomenon of stacking faults upon charging, which significantly stabilizes the reversible large capacity operation. Driving force of the 3D self-ordering is strong long-range cooperative Coulombic interactions between MO_3_ slabs intermediated by ordered vacancies.

## Results

### Stacking faults in Na_2_RuO_3_

**Fig. 1:**
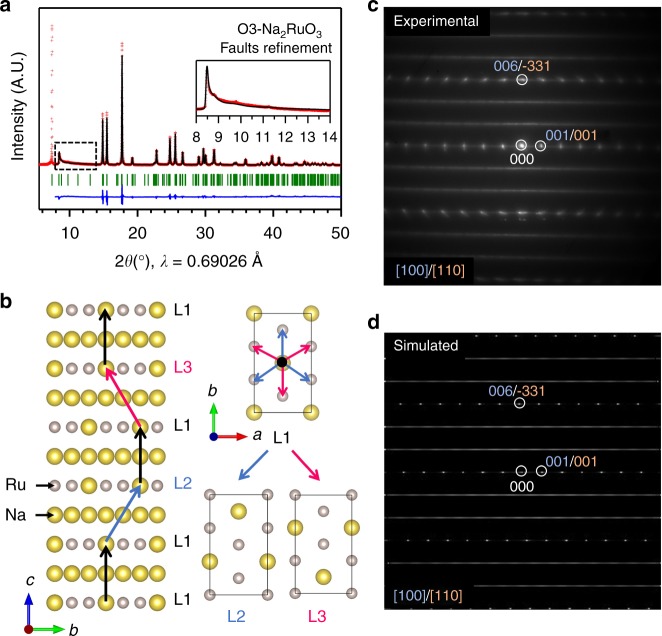
Stacking faults in Na_2_RuO_3_. **a** Observed and calculated (FAULTS refinement) synchrotron XRD patterns of O3-Na_2_RuO_3_ (pristine state). Observed data, the calculated pattern, and the difference between observed and calculated data are shown as plus sign (red), solid line (black) and continuous line (blue), respectively. The positions of Bragg reflections are indicated by vertical tick marks (green). The first diffraction peak has been excluded from the refinement due to important asymmetry that FAULTS does not take into account. The insert is a zoom of the initial superstructure peaks (Warren fall). **b** Representation of the stacking faults in O3-Na_2_RuO_3_, using the FAULTS unit cell described in the text. **c**, **d** Experimental and simulated SAED pattern along the [100] (= <[110]_*C*__2__/__*m*_>) direction, respectively

O3-Na_2_RuO_3_ was synthesized by decomposing Na_2_RuO_4_ at 850 °C for 12 h under Ar atmosphere^20,25^. Figure 1a shows the experimental and calculated synchrotron XRD patterns of O3-Na_2_RuO_3_, in which the most of intense diffraction peaks can be fitted by the usual rhombohedral lattice of O3-AMO_2_ layered oxides^20^. However, the main difficulty hindering an accurate pattern fit lies in the broad nature of some diffraction peaks and diffuse scatterings, which are highlighted by the dashed rectangle in Fig. 1a. Such broadening is typically observed for A_2_MO_3_ with honeycomb ordered [A_1/3_M_2/3_]O_2_ layers, and arises from stacking disorder. These stacking faults can be described by an occasional shift of the [A_1/3_M_2/3_]O_2_ layers perpendicularly to the stacking direction. In fact, in layered materials, the crystal grows perpendicularly to the layer plane. When a nucleation starts in a wrong position, a stacking fault appears while the oxygen packing remains ideal. As a result, the honeycomb ordering of the [A_1/3_M_2/3_]O_2_ layers is maintained but the honeycomb stacking deviates from the ideal sequence (Fig. 1b), which causes the peculiar asymmetric peak broadening (Warren fall) observed in Fig. 1a as well as the diffuse streaks on the selected area electron diffraction (SAED) pattern along the [100]−<110>_*C*2/*m*_ direction (Fig. 1c).

To refine the powder diffraction data for the structure containing stacking faults, a FAULTS analysis, which allows the incorporation of the occurrence probabilities of possible stackings, was conducted^27^. As shown in Fig. 1a (Supplementary Tables 1 and 2), the FAULTS analysis provides a satisfactory description of the superstructure peaks and indicates the occurrence of ~40% stacking faults between the [Na_1/3_Ru_2/3_]O_2_ layers in pristine O3-Na_2_RuO_3_. This result is further supported by the FAULTS-simulated SAED pattern that well reproduces the diffuse streaks observed in the experimental SAED pattern (Fig. 1d).

**Fig. 2:**
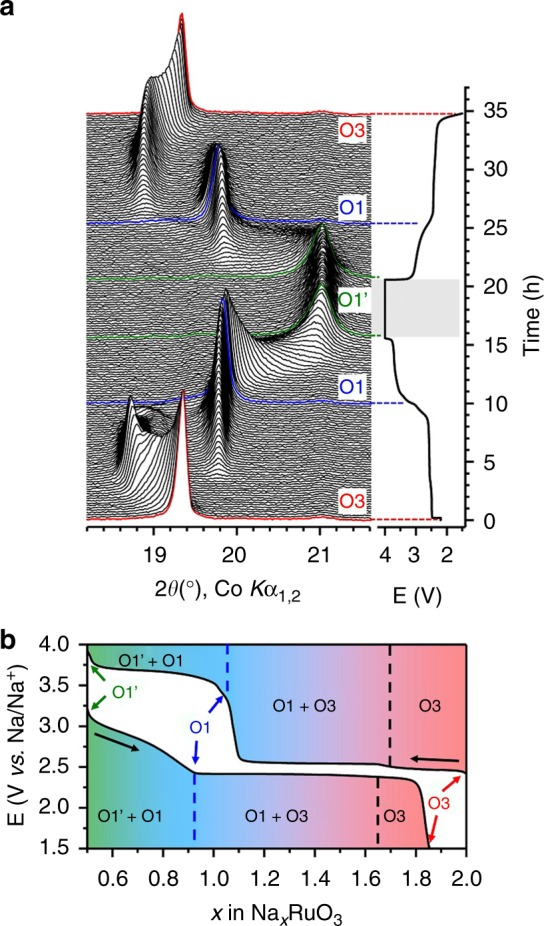
Structural evolution upon charging/discharging Na_2_RuO_3_. **a** XRD patterns recorded in situ during the first cycle of Na_2_RuO_3_ with the corresponding cycling curve. The dashed area corresponds to a potentiostatic break whose aim was to ensure the equilibrium state at 4.0 V before discharging the cell. **b** Phase diagram as determined from the in situ experiment as a function of the sodium content

**Fig. 3:**
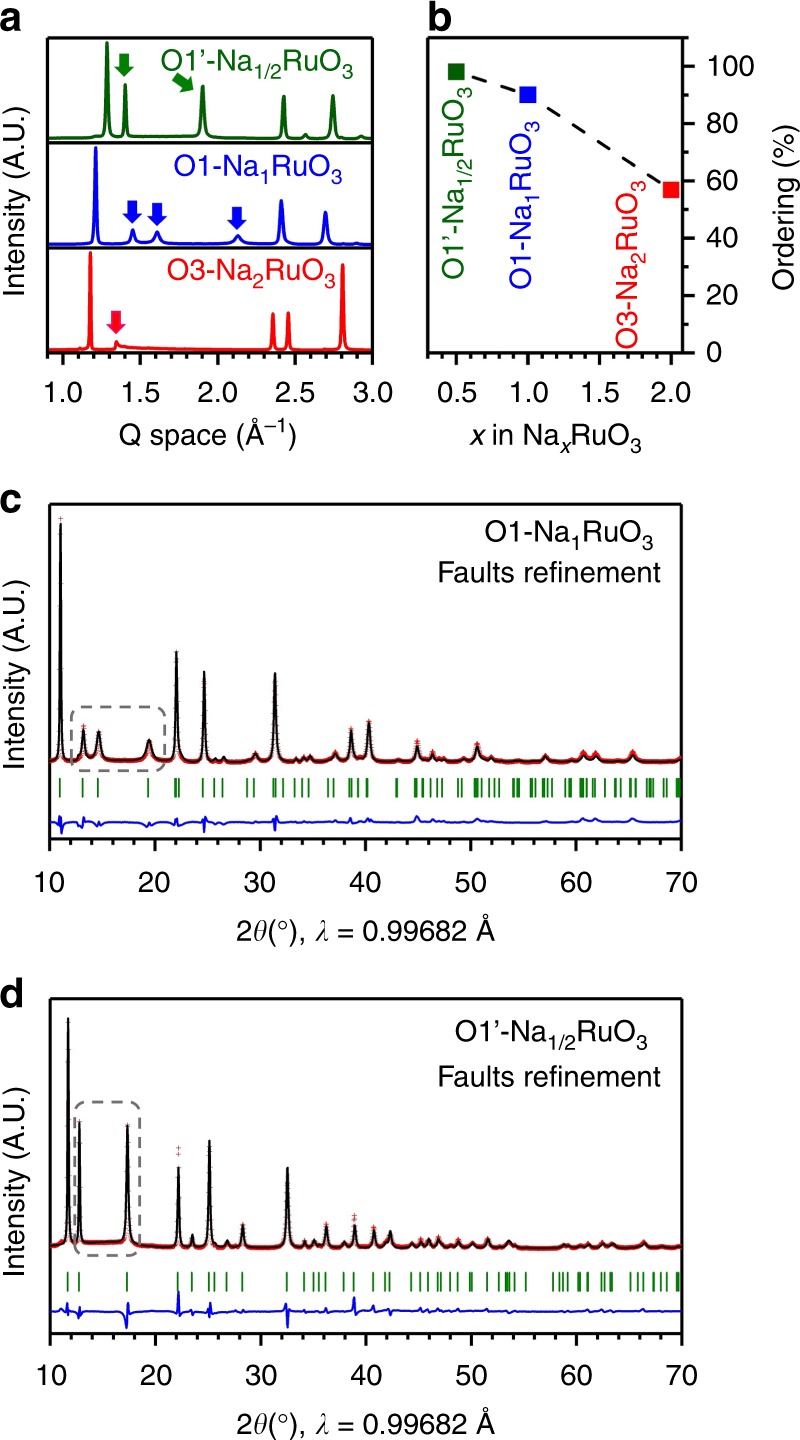
Progressive stacking-fault-depression (self-ordering) upon charging Na_2_RuO_3_. **a** Synchrotron XRD patterns of O3-Na_2_RuO_3_, O1-Na_1_RuO_3_ and O1′-Na_1/2_RuO_3_. Arrows indicate the most intense superstructure peaks. **b** Evolution of the ordered stacking (absence of stacking faults) as a function of the Na content. The error bars are smaller than the mark size. **c**, **d** Observed and calculated (FAULTS refinement) synchrotron XRD patterns of O1-Na_1_RuO_3_ and O1′-Na_1/2_RuO_3_, respectively. Red crosses: experimental, black line: calculated, blue line: difference plot and green bars: Bragg positions in the R$\bar{3}$:h and P$\bar{3}$1*m* space groups for O1-Na_1_RuO_3_ and O1′-Na_1/2_RuO_3_, respectively. The dashed rectangles indicate the most intense superstructure peaks

After evaluation of the stacking faults in pristine O3-Na_2_RuO_3_, we studied the structural evolution of Na_*x*_RuO_3_ during the first charge and discharge using in situ XRD. Figure 2 shows that the phase transformation of Na_*x*_RuO_3_ involves three main phases: O3-Na_*x*_RuO_3_, O1-Na_1_RuO_3_, and O1′-Na_1/2_RuO_3_. (De)sodiation at 2.7 V vs. Na/Na^+^ (1.0<*x*<2.0) mainly proceeds through a two-phase process between O3-Na_*x*_RuO_3_ and O1-Na_1_RuO_3_. As reported previously, O1-Na_1_RuO_3_ has an ilmenite-type structure (ABAB oxide-ions stacking and Na^+^ ions in interlayer octahedral sites) where the honeycomb ordered [Ru_2/3_□_1/3_]O_2_ and [Na_2/3_□_1/3_]O_2_ layers (□: Na^+^ vacancy) stack alternatively (Supplementary Fig. 2)^20^. At the second charging plateau, a new phase (O1′-Na_1/2_RuO_3_) with a shorter interlayer distance (4.91 Å vs. 5.21 Å for O1-Na_1_RuO_3_) appears at the expense of the O1 phase. Its structure was determined from the synchrotron XRD pattern of an electrochemically deintercalated sample, whose Rietveld refinement is presented in Supplementary Fig. 3 and Supplementary Table 3. The diffraction peaks are successfully indexed in a hexagonal lattice with *P*$\bar{3}$1 *m* symmetry with *a* = 5.1876(9) Å and *c* = 4.906(1) Å. As the oxide-ion stacking sequence is ABAB and Na^+^ occupies an octahedral site, we will refer to this phase as O1′-Na_1/2_RuO_3_, where the alternate stacking of the honeycomb ordered [Ru_2/3_□_1/3_]O_2_ and [Na_1/3_□_2/3_]O_2_ layers is maintained. Then, the difference between the O1 and O1′ structures lies in the Na content and on the respective stacking of the [Ru_2/3_□_1/3_]O_2_ layers, which are shifted from one another in O1-Na_1_RuO_3_ while directly stacked in O1′-Na_1/2_RuO_3_ (Supplementary Figs. 2 and 3). It is noteworthy that the superstructure peaks highlighted by the arrows in Fig. 3a exhibit remarkable sharpening upon charging. This suggests that stacking faults tend to disappear, assisted by the adjustable layer gliding involved in the O3→O1′ transition. The FAULTS refinements (Fig. 3c, d and Supplementary Tables 4–6) indicate that the occurrence probability of stacking faults indeed decreases from 40% in O3-Na_2_RuO_3_ to 10% (O1-Na_1_RuO_3_) and then to 2% (O1′-Na_1/2_RuO_3_) (Fig. 3b). Therefore, Na^+^ deintercalation (charging) from Na_2_RuO_3_ involves a self-reorganization process that significantly diminishes the initial amount of stacking faults. Importantly, the stacking faults are reformed after discharging, making this ordering/faulting process reversible, even after several cycles (Supplementary Figs. 4 and 5).

### Self-ordering of stacking faults upon charging Na_2_RuO_3_

**Fig. 4:**
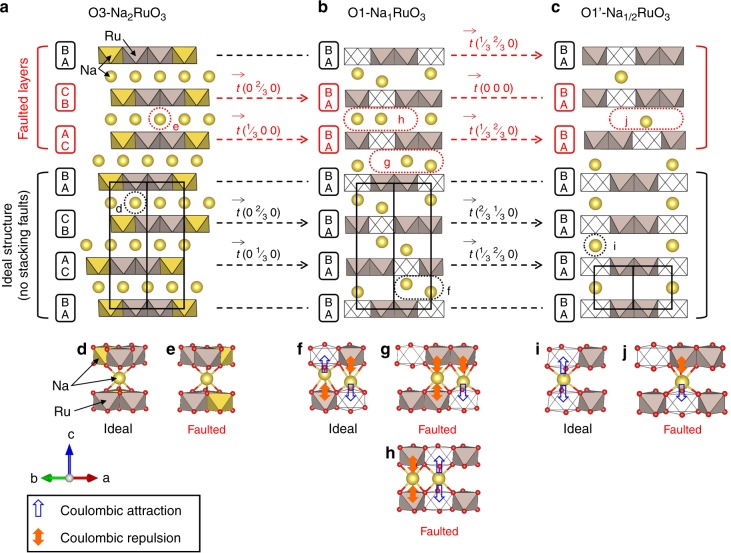
Coulombic forces and resultant stacking–fault–depression (self-ordering) in Na_2−*x*_RuO_3_. Structural representation and projected stacking sequences of the Ru atoms of **a** O3-Na_2_RuO_3_, **b** O1-Na_1_RuO_3_ and **c** O1′-Na_1/2_RuO_3_. The monoclinic cell of O3-Na_2_RuO_3_ has been converted to a pseudo-hexagonal supercell (black lines) for comparison. The transition vectors in the hexagonal supercell from one [Ru_2/3_Na_1/3_]O_2_ or [Ru_2/3_□_1/3_O_2_] layer to another are indicated with respect to the pseudo-hexagonal or hexagonal cells. Note that the transition vectors assigned to faulted layers are a few examples out of many possibilities listed in Supplementary Figs. 5 and 6. **d**‒**j** Comparison of the Na environments in ideal and faulted stacking highlighted with dashed circles in the structures shown in **a**‒**c**

Based on the complete knowledge of the structural evolution from O3-Na_2_RuO_3_ to O1-Na_1_RuO_3_, and then to O1′-Na_1/2_RuO_3_, let us now consider the Coulombic origin of the consolidation on the phase transformation with the effect of gliding vectors *t*^→^ of the [Ru_2/3_Na_1/3_]O_2_ (or [Ru_2/3_□_1/3_]O_2_) layers (Fig. 4a–c). Note that the vectors are given in the pseudo-hexagonal cell (O3-Na_2_RuO_3_) or in the hexagonal cells (O1-Na_1_RuO_3_ and O1′-Na_1/2_RuO_3_; Supplementary Table 7). In O3-Na_2_RuO_3_ (Fig. 4a), the NaO_6_ octahedron in Na layers shares edges with two NaO_6_ and four RuO_6_ octahedra in the adjacent [Ru_2/3_Na_1/3_]O_2_ layers (Fig. 4d). As the stacking faults only impact the stacking of the [Ru_2/3_Na_1/3_]O_2_ layers, the Na^+^ ions in the Na layer have the same local environment (Fig. 4e) and it can be considered that a different stacking is equally probable in O3-Na_2_RuO_3_. However, in O1-Na_1_RuO_3_ consisting of honeycomb ordered [Ru_2/3_□_1/3_]O_2_ and [Na_2/3_□_1/3_]O_2_ layers with the ABAB oxygen packing (Fig. 4b), all the octahedral positions in the slab share faces with the octahedral positions of the interslab space (Supplementary Fig. 6). As a result, the gliding vectors *t*^→^ that shift each layer of O3-Na_2_RuO_3_ to the ones of O1-Na_1_RuO_3_ are ideally adjusted to set each NaO_6_ octahedron in the [Na_2/3_□_1/3_] layers to share faces with a □O_6_ octahedron and a RuO_6_ octahedron in the adjacent [Ru_2/3_□_1/3_]O_2_ layers (Fig. 4f and Supplementary Fig. 7). The stronger attraction of the oxygen atoms surrounding a vacancy on the Na^+^ ion (later referred to as Na^+^−□ Coulombic attraction for simplicity) and the Na^+^−Ru^5+^ Coulombic repulsion cooperatively displace the Na^+^ ion toward □^20^. When assuming the hypothetical stacking faults (Fig. 4g, h), one of two NaO_6_ octahedra is trapped between two RuO_6_ octahedra, where strong Ru^5+^−Na^+^−Ru^5+^ Coulombic repulsions make the faulted structural option unfavorable. Consequently, during the O3 to O1 transformation, slab gliding tends to occur in a way which minimizes the Coulombic energy and as a consequence removes the stacking faults, as described in Fig. 4 and Supplementary Fig. 7. Owing to the three-directional gliding possibilities, most of the stacking faults disappear during the transformation. However, the NaO_6_ octahedron can occasionally be stabilized by the local □−Na^+^−□ Coulombic attraction (Fig. 4h), and some stacking faults may remain in O1-Na_1_RuO_3_.

On the other hand, in O1′-Na_1/2_RuO_3_ (Fig. 4c and Supplementary Fig. 3b), which consists of honeycomb ordered alternating [Ru_2/3_□_1/3_]O_2_ and [Na_1/3_□_2/3_] layers, the NaO_6_ octahedron shares faces with two □O_6_ octahedra in the adjacent [Ru_2/3_□_1/3_]O_2_ layers (Fig. 4c, i). Again with hypothetical stacking faults resulting from *t*^→^ different from the ideal ones shown in Supplementary Fig. 8, the NaO_6_ octahedron in the [Na_1/3_□_2/3_] layer shares faces with □O_6_ and RuO_6_ octahedra, and the local Na^+^-Ru^5+^ Coulombic repulsion prohibits the formation of the faulted structure (Fig. 4j). Therefore, the amount of the stacking faults in Na_*x*_RuO_3_ (1/2 ≤ *x* *≤* 2) continuously diminishes as desodiation proceeds, because the ordered stacking sequences become electrostatically more favorable. As discussed above, no peculiar local environment is stabilized in O3-Na_2_RuO_3_, which allows for stacking faults reformation upon sodiation (Supplementary Figs. 4 and 5).

## Discussion

**Fig. 5:**
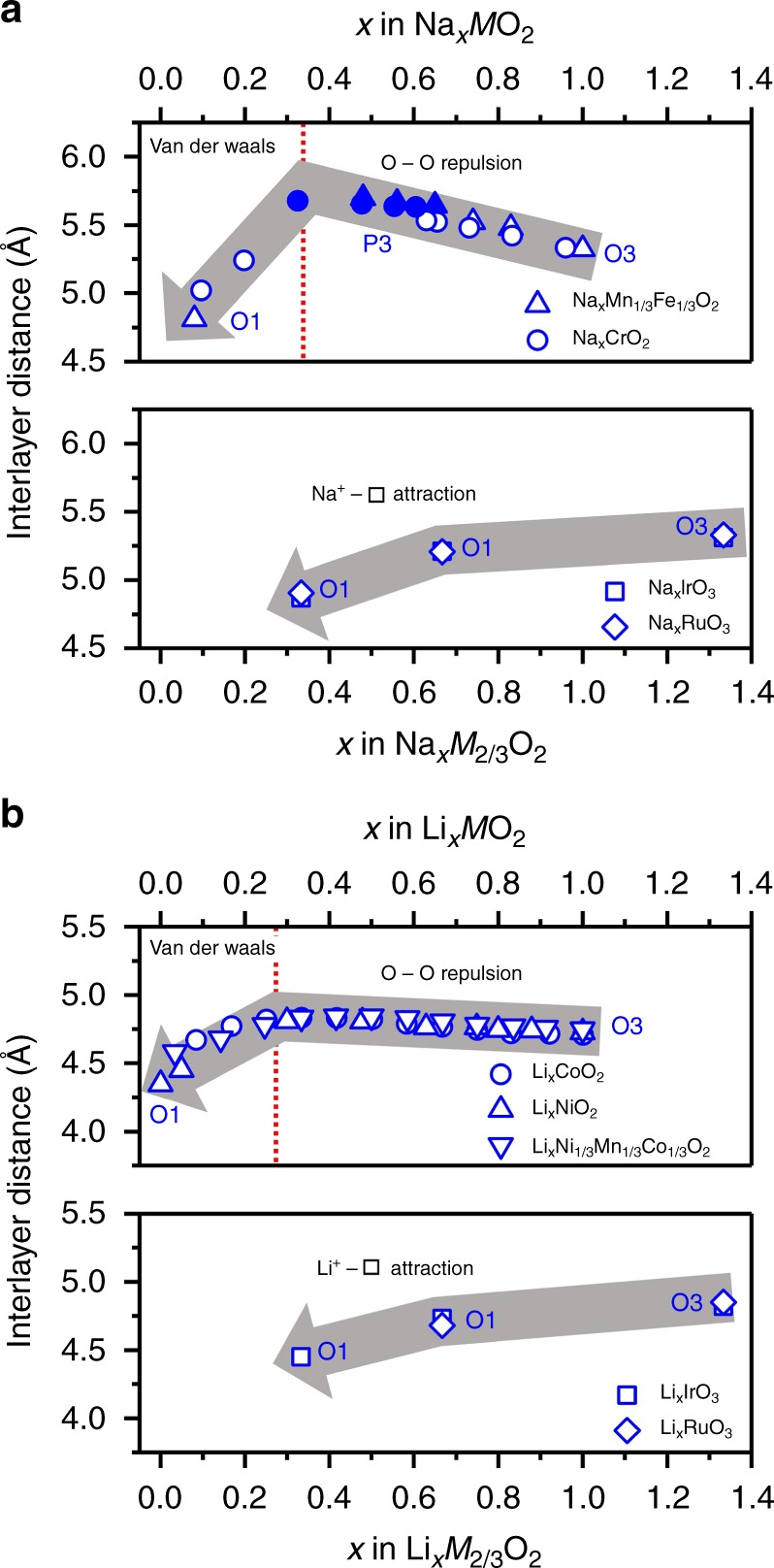
Overviewing dominant forces for phase transformation during A^+^ de-intercalation from AMO_2_ and A_2_MO_3_. Comparison of the general trends in interlayer distance evolution between **a** Na_*x*_MO_2_ (M = Cr^28^ and Fe_2/3_Mn_1/3_^8^) and Na_2_MO_3_ (M = Ru (this study) and Ir^22^) and **b** Li_*x*_MO_2_ (M = Co^4^, Ni^6^ and Ni_1/3_Mn_1/3_Co_1/3_^7^) and Li_*x*_MO_3_ (M = Ru^17^ and Ir^18^)

The aforementioned importance of the attractive A^+^−□ and repulsive A^+^−M^5+^ Coulombic interactions can lead to more general discussions on the phase stability of A_2_MO_3_ and AMO_2_ upon charging. Figure 5a shows the experimental evolution of the interlayer distance upon charging various Na_*x*_MO_2_ (M = 3d)^8,28^ and Na_2_MO_3_ (M = 4d, 5d)^22^ compounds as a function of the sodium content. Figure 5b shows the similar plots for the lithium analogs, LiMO_2_ and Li_2_MO_3_^4,6,7,17,18^. It is noteworthy that all AMO_2_ (A = Li, Na and M = 3d) and all A_2_MO_3_ (A = Li, Na and M = 4d, 5d) respectively follow the same tendencies.

As mentioned in the introduction, the phase transformation of *A*_*x*_MO_2_ is dominated by the competing O−O Coulombic repulsion (0.4 < *x* < 1.0) and van der Waals attraction (0 < *x* < 0.4) between adjacent MO_2_ layers^4–8^, which initiates the increase of the interlayer distance (0.4 < *x* < 1.0) followed by its abrupt decrease upon deeper charging (0 < *x* < 0.4). Structural evolution in the “Coulombic domain” with large A^+^ content (0.4 < *x* < 1.0) are highly reversible and is the basis of commercial positive electrode materials such as LiCoO_2_ and Li[Ni_1-*y*-*z*_Mn_*y*_Co_*z*_]O_2_, while the “van der Waals domain” with smaller A^+^ content (0 < *x* < 0.4) marks the limit of reversibility of many practical layered oxides as a result of cation migration, spinel transformation, crack formation, and delamination/exfoliation^8–13^.

On the contrary, the highly ordered nature of the [Ru_2/3_□_1/3_]O_2_ layers of A_*x*_MO_3_ (M = Ru or Ir) triggers a progressive gain in A^+^−□ Coulombic energy upon A^+^ extraction as the remaining A^+^ ions cooperatively act as robust pillars between adjacent [Ru_2/3_□_1/3_]O_2_ layers to prevent structural collapse. The slight decrease of the interlayer distance is driven by Coulombic A^+^−□ attractive forces which are strong enough to induce the slab glidings, O3 → O1 → O1′, forming a more-ordered structure upon charging. It is this situation that realizes reversible charge–discharge reactions in ordered A_2_MO_3_ over a wide compositional domain of the guest ion A^+^.

In summary, we identified a spontaneous reorganization of the stacking faults in Na_*x*_RuO_3_, a model material to understand oxygen-redox reactions in layered oxides for large-capacity battery electrodes. In particular, the progressive ordering upon charging process is a general phenomenon to A_2_MO_3_ (A = Li, Na and M = 4d, 5d) materials and is induced by the cooperative effect of maximizing the A^+^−□ Coulombic attraction and minimizing the A^+^−M^5+^ Coulombic repulsion, which significantly enlarges the reversible operation range of layered oxides. Complementarily to our previous work, in addition to generate redox-active “orphaned” nonbonding oxygen 2*p* orbitals to “activate” additional oxygen-redox reactions, honeycomb ordering of M and □ contribute to “stabilizing” reversible phase transformation. In this regard, the importance of the overall material design that includes ordered vacancies with its neutral charge to attract alkali cations was highlighted. By establishing proper ways to control the stacking faults^29–31^ or vacancies^32^, the concept of progressive ordering upon charging may be extended to stabilize other related compounds^33^.

## Methods

### Synthesis of Na_2_RuO_3_

Na_2_RuO_3_ was prepared according to the literature^25^. First, Na_2_RuO_4_ is prepared by mixing stoichiometric amounts of Na_2_O_2_ (Sigma-Aldrich) and RuO_2_ (Kanto chemicals). Pellets are then made and introduced in a tubular furnace to be annealed at 650 °C for 12 h under O_2_ atmosphere. After the synthesis, Na_2_RuO_4_ is grinded and shaped into pellets again to be thermally decomposed into Na_2_RuO_3_ at 850 °C for 12 h under Ar atmosphere. After cooling down to room temperature, the sample is introduced in an Ar filled glovebox.

### Characterization

The synchrotron XRD patterns were recorded at Aichi Synchrotron Radiation Center (Aichi-SR, O3-Na_2_RuO_3_), and Photon Factory at High Energy Accelerator Research organization (KEK-PF, BL-8B) or SPring-8 (O1-Na_1_RuO_3_ and O1′-Na_1/2_RuO_3_, beamline 02B2). All samples were protected from air exposure during the measurement. Rietveld refinement was performed using Jana2006^34^. Analyses of the stacking faults in the materials were carried out using the FAULTS software^27^. The crystal structures were drawn using VESTA^35^. Selected Area Electron Diffraction (SAED) patterns were recorded using an electron microscope (HF-3000S; Hitachi Ltd. and Titan Cubed; FEI Co.) operated at 300 kV. The camera length for SAED was calibrated with a Si crystal.

### Electrochemistry

Electrochemical measurements were carried out in Na_2_RuO_3_/electrolyte/Na half-cells assembled in CR2032 type coin cells. The electrolyte was 1 mol/L NaPF_6_ in EC:DEC (1:1) purchased from Chameleon Reagent. Positive electrodes were prepared by coating a slurry made of active material (80 wt%) mixed with acetylene black (10 wt%) and polyvinylidene (10 wt%) in NMP onto Al foil. Sixteen-mm-diameter electrodes were cut after drying for one night under vacuum at 120 °C. The positive and negative electrodes were separated by a layer of Whatman glass fiber separator soaked with electrolyte. The galvanostatic curve was recorded and controlled using a TOSCAT-3100 battery tester. The charge/discharge rate was C/10 which corresponds to the (de)intercalation of 1 Na^+^ per Na_x_RuO_3_ in 10 hours. The O1-Na_1_RuO_3_ and O1′-Na_1/2_RuO_3_ samples were prepared by cycling sintered pellets (diameter 10 mm, weight ≈10–15 mg) of O3-Na_2_RuO_3_ at C/50 (one Na^+^ exchanged in 50 h) until a given voltage (3.07 V and 4.0 V vs. Na/Na^+^ for O1-Na_1_RuO_3_ and O1′-Na_1/2_RuO_3_, respectively). The recovered materials were then washed 3 to 5 times with dimethyl carbonate in the glovebox before sending to the synchrotron facility.

In situ X-ray diffraction. In situ XRD was carried out in operando using an in situ cell purchased from Bruker on a Bruker-AXS D8 ADVANCE (Co *K*α radiation) in 0.02^o^ steps over the 2*θ* range of 17–25^o^ at a C/10 rate. The cell configuration is similar to the one described above except for the positive electrode that consisted in a mixture of Na_2_RuO_3_ (85 wt%), acetylene black (10 wt%), and polytetrafluoroethylene (5 wt%). Two layers of glass fiber separators were used to prevent dendrite formation.

## Supplementary information

Supplementary Information

Peer Review File

## Data Availability

The whole datasets are available from the corresponding author on request.
