# Supplementary material for: Coulombic self-ordering upon charging a large-capacity layered cathode material for rechargeable batteries
Source: Nat Commun. 2019 May 16;10:2185. doi: 10.1038/s41467-019-09409-1 (PMC6522540; doi:10.1038/s41467-019-09409-1)
Supplement: Supplementary file 1 — Supplementary Information [file 41467_2019_9409_MOESM1_ESM.pdf]

Supplementary information

Coulombic self-ordering upon charging a large-capacity layered battery electrode

Mortemard de Boisse et al.

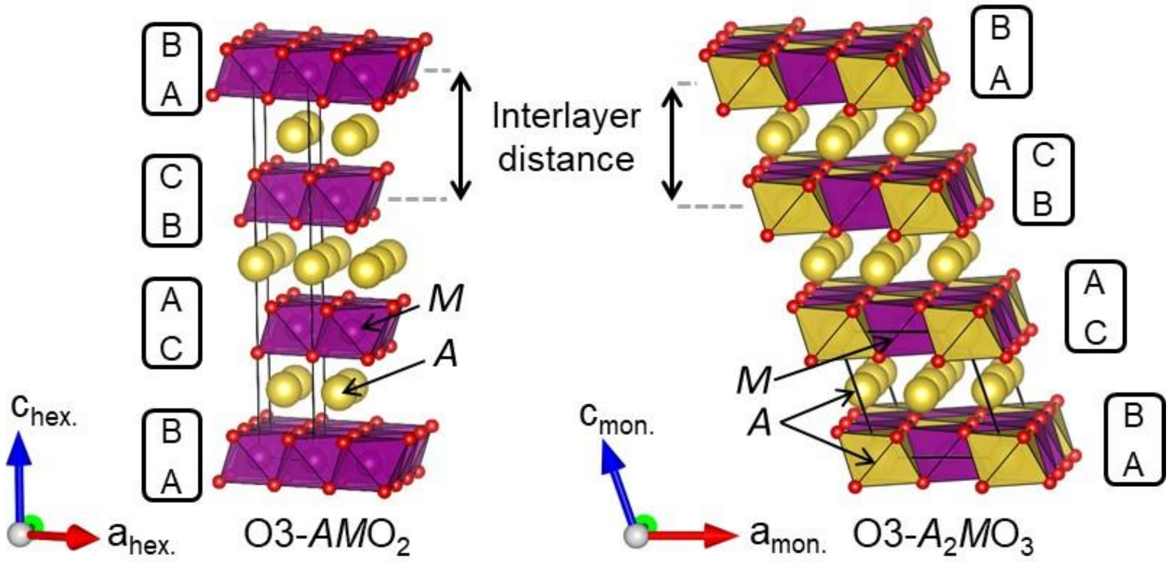

Supplementary Figure 1. Ideal structural representations of O3-AMO_2 (left) and O3-A_2MO_3 (right). O3-AMO_2 is generally described using an hexagonal *R*-3*m* unit cell, while a monoclinic *C*2/*m* is used to take into account the symmetry reduction arising from the honey-comb ordering of *A* and *M* cations in the $[A_{1/3}M_{2/3}]O_2$ layers.

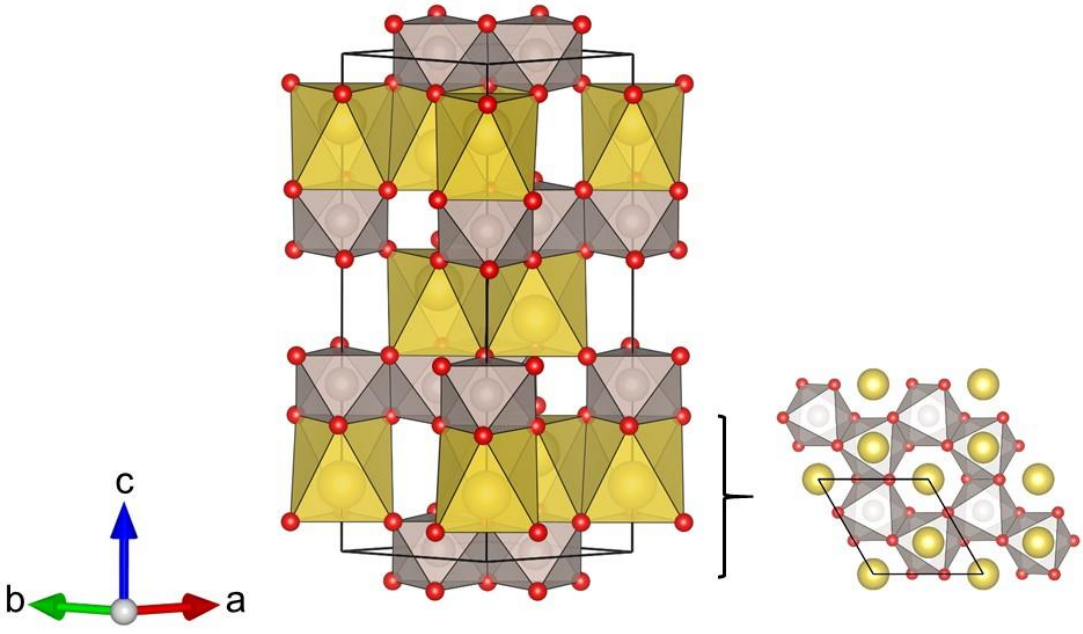

Supplementary Figure 2. Representation of the O1-Na_1RuO_3 structure and highlight of the honeycomb ordering in the $[Ru_{2/3}\square_{1/3}]O_2$ and $[Na_{2/3}\square_{1/3}]O_2$ layers.

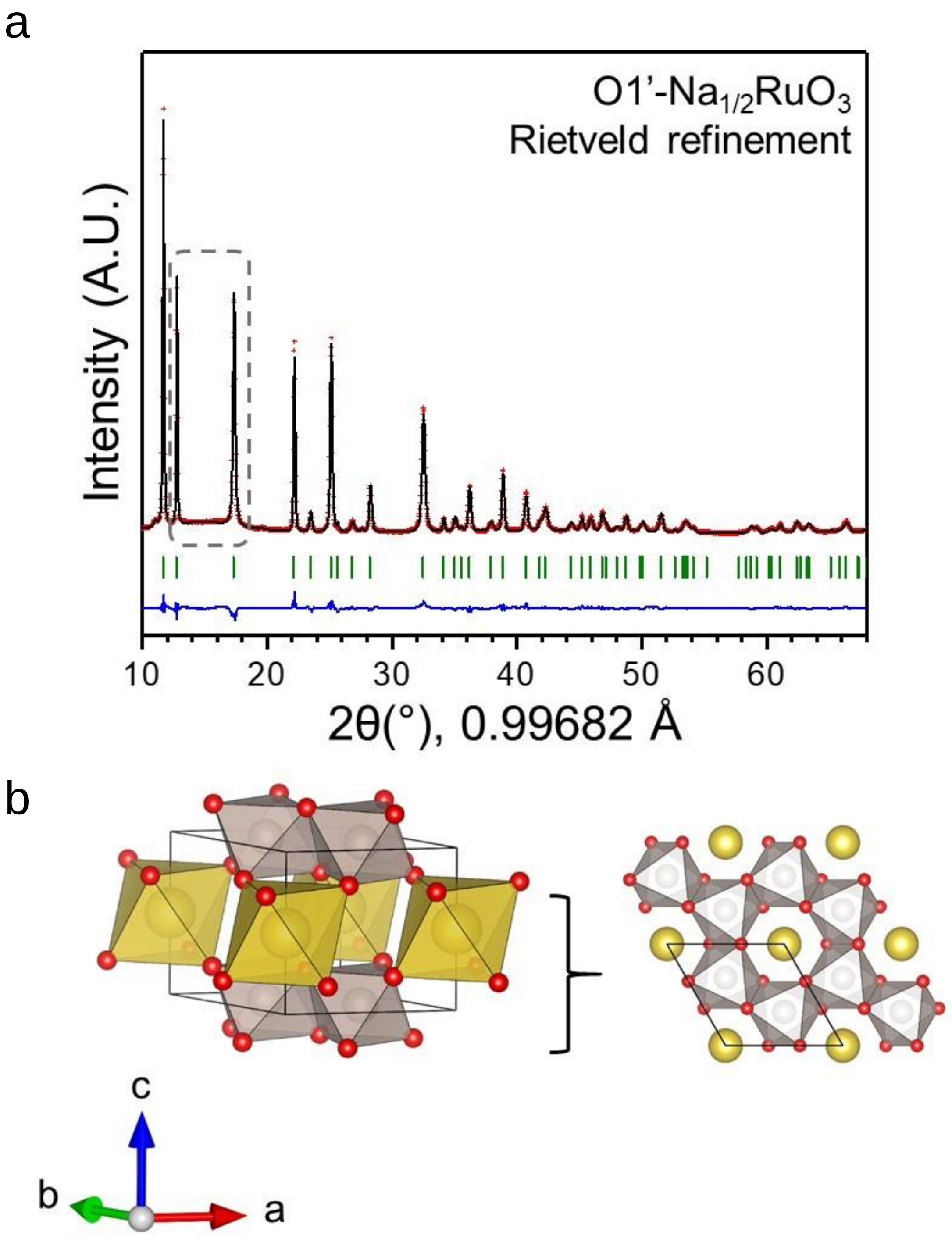

Supplementary Figure 3. Structure of O1'-$Na_{1/2}RuO_3$. a, Observed and calculated (Rietveld refinement) synchrotron XRD patterns of O1'-$Na_{1/2}RuO_3$. Red crosses: experimental, black line: calculated, blue line: difference plot and green bars: Bragg positions. The dashed rectangle indicates the more intense super-structure peaks. **b**, Representation of the O1'-$Na_{1/2}RuO_3$ structure and highlight of the honeycomb ordering in the $[Ru_{2/3}\square_{1/3}]O_2$ and $[Na_{1/3}\square_{2/3}]O_2$ layers.

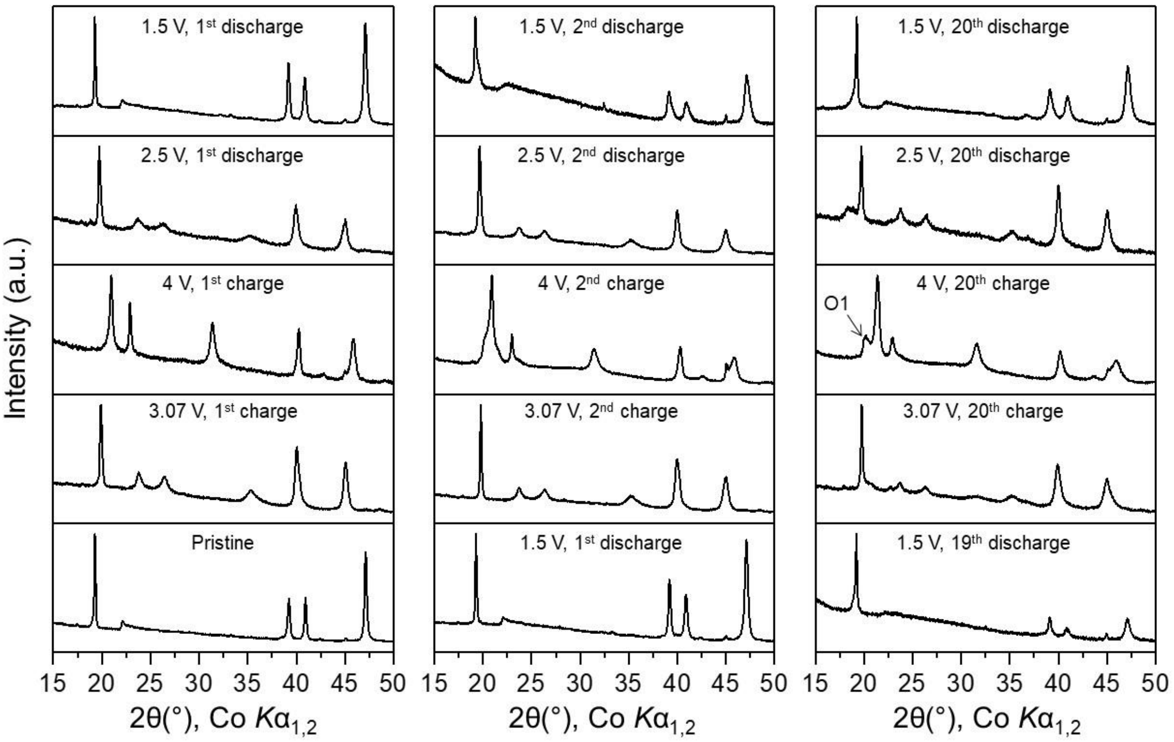

Supplementary Figure 4. ***Ex situ*** **XRD patterns during cycling Na_2RuO_3.** The electrodes were charged to 3.07 V (O1-Na_1RuO_3) and 4 V (O1'-$Na_{1/2}RuO_3$) and discharged to 2.5 V (O1-Na_1RuO_3) and 1.5 V (O3-Na_2RuO_3) during the 1st, 2nd, and 20th cycles. A small fraction of the O1 phase remains at the 20th charged state presumably due to reaction inhomogeneity, making FAULT analysis difficult.

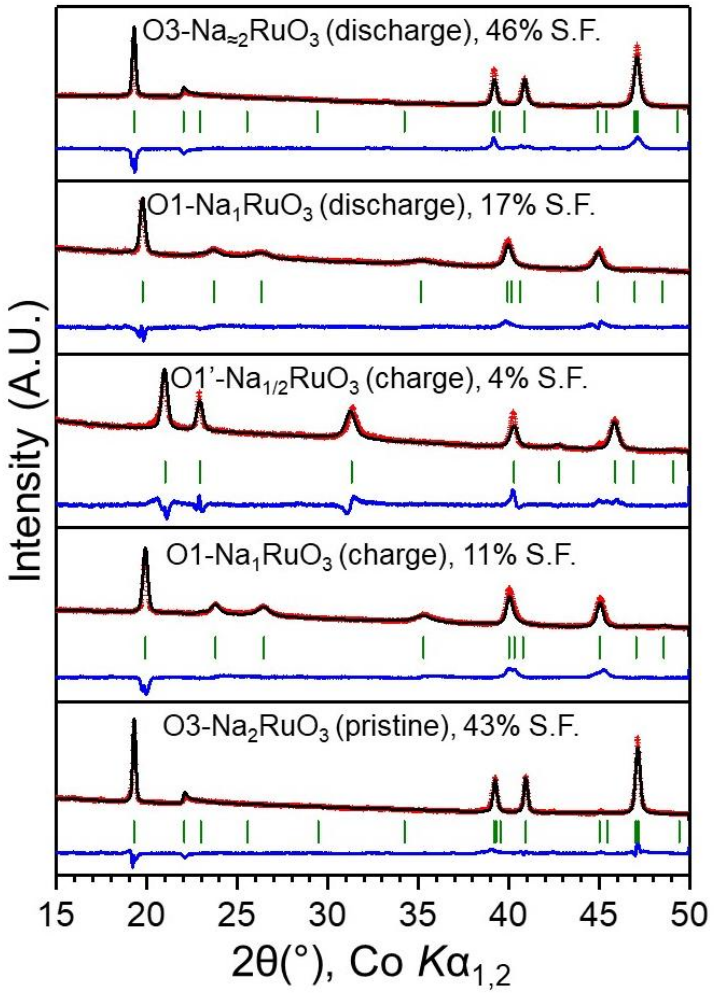

Supplementary Figure 5. Estimation of the amount of stacking faults in Na_2RuO_3 during cycling. Observed and calculated (FAULTS refinements) *ex situ* XRD patterns collected on Al-coated electrodes of O3-Na_2RuO_3, O1-Na_1RuO_3 and O1'-$Na_{1/2}RuO_3$ during charge and discharge with corresponding estimated stacking faults (S.F.) amounts. Due to the few number of peaks observed in the selected range atomic parameters were kept identical to the ones determined from the synchrotron XRD data.

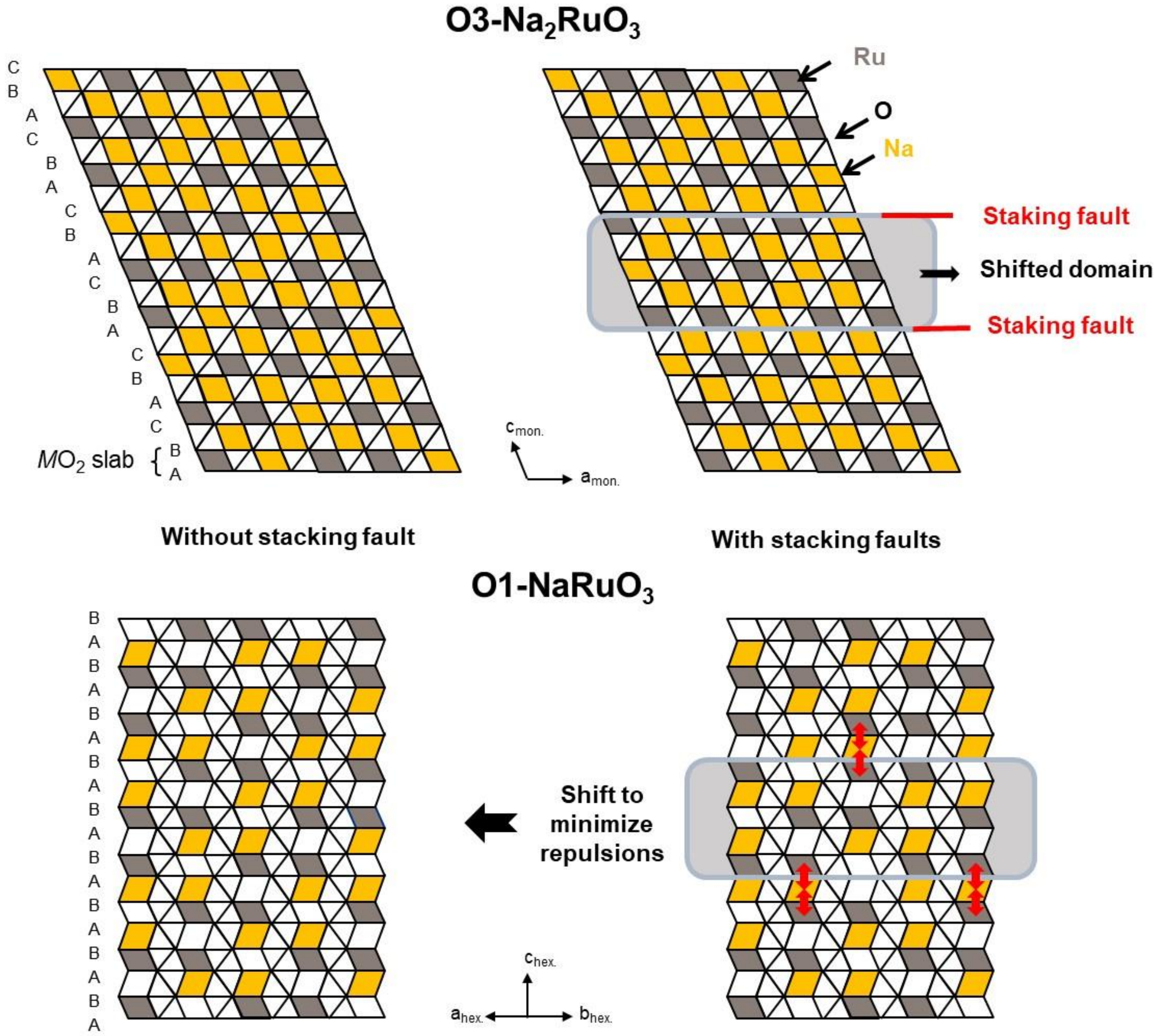

Supplementary Figure 6. Comparison between the ideal and faulted structures of O3-Na_2RuO_3 (top) and O1-Na_1RuO_3 (bottom). The octahedral sites are represented by a parallelogram and the tetrahedral sites by a triangle. The oxygen atoms are localized at the corners between the octahedra and the tetrahedra. The AO_6 octahedra share edges with the MO_6 octahedra in the O3 structure while they share faces in the O1 structure. The red arrows represent the strong Ru^{5+} – Na^{+} – Ru^{5+} repulsions in O1-Na_1RuO_3.

Layer number	Type	O3-Na_2RuO_3	$\vec{t}$ O3 → ideal O1	O1-Na_1RuO_3
**Layer 1**	Ideal	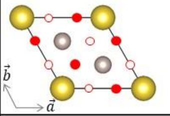	0 0 0	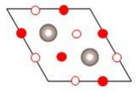
**Layer 2**	Ideal	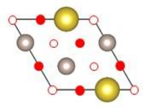	0 ⅓ 0	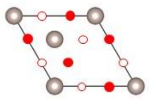
	Faulted	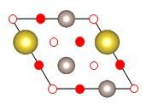	-⅓ -⅓ 0	
	Faulted	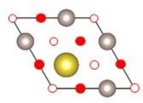	⅓ 0 0	
**Layer 3**	Ideal	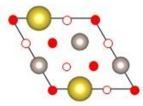	0 -⅓ 0	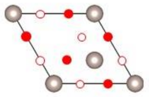
	Faulted	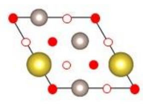	⅓ ⅓ 0	
	Faulted	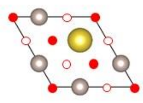	-⅓ 0 0	

Layer number	Type	O3-*AMO*$_2$	$\vec{t}$ O3 → ideal O1	O1-A_0MO_2
**Layer 1**	Ideal	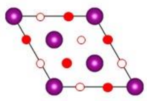	0 0 0	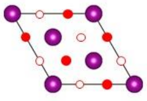
**Layer 2**	Ideal	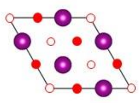	0 ⅓ 0<br>-⅓ -⅓ 0<br>⅓ 0 0	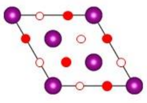
**Layer 3**	Ideal	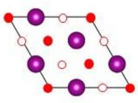	0 -⅓ 0<br>⅓ ⅓ 0<br>-⅓ 0 0	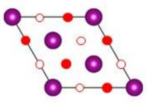

Supplementary Figure 7. Possible slab glides upon charging Na_2RuO_3 and AMO_2. (top) Possible transition vectors $\vec{t}$ from the ideal and faulted layers of O3-Na_2RuO_3 to the ideal layers of O1-Na_1RuO_3. The yellow spheres represent the Na atoms and the grey spheres represent the Ru atoms. The red circles represent the position of the O atoms located above (full circles) and bellow (open circles) the Ru atoms. (bottom) Comparison with the transition vectors from O3-AMO_2 to O1-A_0MO_2. Every distinct transition vector in from O3-Na_2RuO_3 appear here equivalent. The purple spheres represent the *M* atoms and the red circles represent the O network. The representation is given in the (ab) plane of the pseudo-hexagonal or hexagonal cells, projected along $c_{hex.}$.

Layer number	Type	$O1\text{-}Na_1RuO_3$	$\vec{t}$ O1 → ideal O1’	$O1'\text{-}Na_{1/2}RuO_3$
**Layer 1**	Ideal	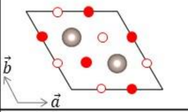	0 0 0	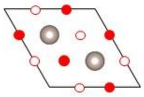
**Layer 2**	Ideal	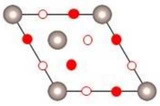	⅓ -⅓ 0	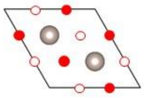
	Faulted	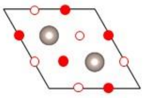	0 0 0	
	Faulted	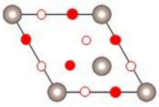	-⅓ ⅓ 0	
**Layer 3**	Ideal	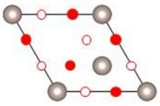	-⅓ ⅓ 0	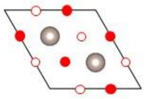
	Faulted	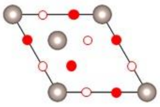	⅓ -⅓ 0	
	Faulted	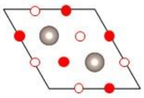	0 0 0	

Supplementary Figure 8. Possible slab glides upon charging Na_1RuO_3. Possible transition vectors $\vec{t}$ from the ideal and faulted layers of O1-Na_1RuO_3 to the ideal layers of O1’-$Na_{1/2}RuO_3$. The grey spheres represent the Ru atoms. The red circles represent the position of the O atoms located above (full circles) and bellow (open circles) the Ru atoms. The representation is given in the (ab) plane of the hexagonal cells, projected along $c_{hex.}$.

Supplementary Table 1. Structural parameters, atomic parameters and transition vectors calculated (FAULTS refinement) from the synchrotron XRD pattern of O3-Na_2RuO_3. Constraints were used during the refinement with respect to the symmetry operations of space group *C*2/*m*.

O3-Na_2RuO_3 (FAULTS refinement)

a_{FAULTS} = 5.40395(2) Å, b_{FAULTS} = 9.36861(3) Å, c_{FAULTS} = 5.34073(2) Å.

α_{FAULTS} = 90°, β_{FAULTS} = 90°, γ_{FAULTS} = 90°.

R_p = 9.8 %

	Atom	x/a_{FAULTS}	y/b_{FAULTS}	z/c_{FAULTS}	ADP (Å^2)	Occupancy
**Layer 1 = 2 = 3**	**Na(1)**	1/2	1/2	0	0.5	1.0
	**Na(2)**	0	0	0	0.5	1.0
	**Ru(1)**	0	1/3	0	0.5	1.0
	**Ru(2)**	0	2/3	0	0.5	1.0
	**Ru(3)**	1/2	1/6	0	0.5	1.0
	**Ru(4)**	1/2	5/6	0	0.5	1.0
	**O(1)**	0.1846(2)	0.8140(1)	0.2001(2)	0.8	1.0
	**O(2)**	0.1846(2)	0.1860(1)	0.2001(2)	0.8	1.0
	**O(3)**	0.1400(2)	1/2	0.2050(2)	0.8	1.0
	**O(4)**	0.6400(2)	0	0.2050(2)	0.8	1.0
	**O(5)**	0.6846(2)	0.3140(1)	0.2001(2)	0.8	1.0
	**O(6)**	0.6846(2)	0.6860(1)	0.2001(2)	0.8	1.0
	**O(7)**	0.3154(2)	0.6860(1)	-0.2001(2)	0.8	1.0
	**O(8)**	0.3154(2)	0.3140(1)	-0.2001(2)	0.8	1.0
	**O(9)**	0.3600(2)	0	-0.2050(2)	0.8	1.0
	**O(10)**	0.8154(2)	0.8140(1)	-0.2001(2)	0.8	1.0
	**O(11)**	0.8154(2)	0.1860(1)	-0.2001(2)	0.8	1.0
	**O(12)**	0.8600(2)	1/2	-0.2050(2)	0.8	1.0
**Layer 4**	**Na(1)**	1/2	2/3	0	1.0	1.0
	**Na(2)**	1/2	1/3	0	1.0	1.0
	**Na(3)**	1/2	0	0	1.0	1.0
	**Na(4)**	0	5/6	0	1.0	1.0
	**Na(5)**	0	1/2	0	1.0	1.0
	**Na(6)**	0	1/6	0	1.0	1.0

	Transition	x/a_{FAULTS}	y/b_{FAULTS}	z/c_{FAULTS}	Probability	Type
From L1	L1 → L4	-0.1680(1)	0	1/2	1	Expected
From L2	L2 → L4	-0.1680(1)	0	1/2	1	Expected
From L3	L3 → L4	-0.1680(1)	0	1/2	1	Expected
From L4	L4 → L1	-0.1680(1)	0	1/2	0.5683(7)	Expected
	L4 → L2	-0.1680(1)	1/3	1/2	0.2159(7)	Fault
	L4 → L3	-0.1680(1)	2/3	1/2	0.2159(7)	Fault

Supplementary Table 2. Structural and atomic parameters of the average unit cell of O3-Na_2RuO_3 deduced from the FAULTS refinement. Space group: *C*2/*m* (#12); *a* = 5.40395 Å; *b* = 9.36861 Å; c = 5.63838 Å and β = 108.7 °

Atom	Site	Fractional coordinates			Occupancy	ADP (Å^2)
Na(1)	2*a*	0	0	0	1.0	0.5
Na(2)	2*d*	0	1/2	1/2	1.0	1.0
Na(3)	4*h*	0	1/6	1/2	1.0	1.0
Ru	4*g*	0	1/3	0	1.0	0.5
O(1)	*4*i	0.7087	0	0.2050	1.0	0.8
O(2)	8*j*	0.2517	0.1860	0.2001	1.0	0.8

Supplementary Table 3. Structural and atomic parameters deduced from the Rietveld refinement of O1'-$Na_{1/2}RuO_3$. Space group: $P\bar{3}1m$ (#162); a = 5.1910(2) Å; c = 4.9002(6) Å; R_B = 2.97%; R_{wp} = 4.73 %.

Atom	Site	Fractional coordinates			Occupancy	ADP (Å^2)
Na	1*a*	0	0	1/2	1.0	2.0(1)
Ru	2*d*	1/3	2/3	0	1.0	1.41(2)
O	6*k*	0.3725(6)	0	0.7873(7)	1.0	1.0(1)

Supplementary Table 4. Structural parameters, atomic parameters and transition vectors calculated (FAULTS refinement) from the synchrotron XRD pattern of O1-Na_1RuO_3. The considered cell is similar to that previously reported for O1-Na_1RuO_3. Constraints were used during the refinement with respect to the symmetry operations of space group $R\bar{3}$:h.

O1-Na_1RuO_3 (FAULTS refinement)

$a_{FAULTS} = b_{FAULTS}$ = 5.24235(1) Å, c_{FAULTS} = 15.67141(7) Å.

α_{FAULTS} = 90°, β_{FAULTS} = 90°, γ_{FAULTS} = 120°.

R_p = 9.08 %

	Atom	x/a_{Faults}	y/b_{Faults}	z/c_{Faults}	ADP ($Å^2$)	Occupancy
**Layer 1 = 2 = 3**	**Ru(1)**	2/3	1/3	-0.00245(2)	0.5	1.0
	**Ru(2)**	1/3	2/3	0.00245(2)	0.5	1.0
	**O(1)**	0.3535(1)	-0.004(3)	-0.06657(1)	0.8	1.0
	**O(2)**	0.6487(1)	0.6464(1)	-0.06657(1)	0.8	1.0
	**O(3)**	0.000(3)	0.3513(1)	-0.06657(1)	0.8	1.0
	**O(4)**	0.3513(1)	0.3535(1)	0.06657(1)	0.8	1.0
	**O(5)**	0.004(3)	0.6487(1)	0.06657(1)	0.8	1.0
	**O(6)**	0.6464(1)	0.000(3)	0.06657(1)	0.8	1.0
**Layer 4**	**Na(1)**	0	0	0.1344(1)	1.0	1.0
	**Na(2)**	2/3	1/3	0.1989(1)	1.0	1.0
**Layer 5**	**Na(1)**	0	0	0.150(4)	1.0	1.0
	**Na(2)**	2/3	1/3	0.164(3)	1.0	1.0
**Layer 6**	**Na(1)**	0	0	0.177(3)	1.0	1.0
	**Na(2)**	2/3	1/3	0.154(4)	1.0	1.0

	Transition	x/a_{FAULTS}	y/b_{FAULTS}	z/c_{FAULTS}	Probability	Type
From L1	L1 → L4	0	0	0	0.8904(1)	Expected
	L1 → L5	0	0	0	0.0548(1)	Fault
	L1 → L6	0	0	0	0.0548(1)	Fault
From L2	L2 → L4	0	0	0	0.8904(1)	Expected
	L2 → L5	0	0	0	0.0548(1)	Fault
	L2 → L6	0	0	0	0.0548(1)	Fault
From L3	L3 → L4	0	0	0	0.8904(1)	Expected
	L3 → L5	0	0	0	0.0548(1)	Fault
	L3 → L6	0	0	0	0.0548(1)	Fault
From L4	L4 → L1	2/3	1/3	1/3	1.0	Expected
From L5	L5 → L2	0	0	0.3334(1)	1.0	Expected
From L6	L6 → L3	1/3	2/3	0.3334(1)	1.0	Expected

Supplementary Table 5. Structural and atomic parameters of the average unit cell of O1-Na_1RuO_3 deduced from the FAULTS refinement. Space group: $R\bar{3}$:h (#148); *a* = 5.22701(2) Å and *c* = 15.63762(4) Å.

**Atom**	**Site**	**Fractional coordinates**			**Occupancy**	**ADP (Å^2)**
Na	6*c*	0	0	0.1344(1)	1.0	1.0
Ru	6*c*	1/3	2/3	0.00245(2)	1.0	0.5
O	18*f*	0.004(3)	0.6487(1)	0.06657(1)	1.0	0.8

Supplementary Table 6. Structural parameters, atomic parameters and transition vectors calculated (FAULTS refinement) from the synchrotron XRD pattern of O1'-$Na_{1/2}RuO_3$. The considered cell is similar to that reported in Table S3. Constraints were used during the refinement with respect to the symmetry operations of space group $P\bar{3}1m$. The few number of slabs impacted by the stacking faults prevented from refining the Na position in the faulted layers.

O1'-$Na_{1/2}RuO_3$ (FAULTS refinement)
$a_{FAULTS} = b_{FAULTS}$ = 5.19308(1) Å, c_{FAULTS} = 4.90314(1) Å.
α_{FAULTS} = 90°, β_{FAULTS} = 90°, γ_{FAULTS} = 120°.
R_p = 7.64 %

	**Atom**	x/a_{FAULTS}	y/b_{FAULTS}	z/c_{FAULTS}	**ADP (Å^2)**	**Occupancy**
**Layer 1 = 2 = 3**	**Ru(1)**	2/3	1/3	0	0.5	1.0
	**Ru(2)**	1/3	2/3	0	0.5	1.0
	**O(1)**	0.61826(2)	0.61826(2)	0.78789(1)	0.8	1.0
	**O(2)**	0.38174(2)	0	0.78789(1)	0.8	1.0
	**O(3)**	0	0.38174(2)	0.78789(1)	0.8	1.0
	**O(4)**	0.38174(2)	0.38174(2)	0.21211(1)	0.8	1.0
	**O(5)**	0.61826(2)	0	0.21211(1)	0.8	1.0
	**O(6)**	0	0.61826(2)	0.21211(1)	0.8	1.0
**Layer 4**	**Na(1)**	0	0	1/2	1.0	1.0
**Layer 5**	**Na(1)**	0	0	1/2	1.0	1.0
**Layer 6**	**Na(1)**	0	0	1/2	1.0	1.0

	**Transition**	x/a_{FAULTS}	y/b_{FAULTS}	z/c_{FAULTS}	**Probability**	**Type**
From L1	L1 → L4	0	0	0	0.98100(1)	Expected
	L1 → L5	0	0	0	0.00950(1)	Fault
	L1 → L6	0	0	0	0.00950(1)	Fault
From L2	L2 → L4	0	0	0	0.98100(1)	Expected
	L2 → L5	0	0	0	0.00950(1)	Fault
	L2 → L6	0	0	0	0.00950(1)	Fault
From L3	L3 → L4	0	0	0	0.98100(1)	Expected
	L3 → L5	0	0	0	0.00950(1)	Fault
	L3 → L6	0	0	0	0.00950(1)	Fault
From L4	L4 → L1	0	0	1/3	1.0	Expected
From L5	L5 → L2	2/3	1/3	1/3	1.0	Expected
From L6	L6 → L3	1/3	2/3	1/3	1.0	Expected

Supplementary Table 7. Comparison between the cell parameters, symmetries and number of MO_2 layers per cell of O3-Na_2RuO_3, O1-Na_1RuO_3 and O1'-$Na_{1/2}RuO_3$.

	Symmetry	Space group	Cell parameters	Number of MO_2 layers per unit cell
**O3-Na_2RuO_3**	Monoclinic	$C2/m$	a = 5.40395(2) Å<br>b = 9.36861(3) Å<br>c = 5.63838(2) Å<br>β = 108.7 °	1
	Pseudo-hexagonal	-	a = 5.40395(2) Å<br>c = 16.0222(1) Å	3
**O1-Na_1RuO_3**	Hexagonal	$R\bar{3}:h$	a = 5. 24235 (2) Å<br>c = 15.67141(7) Å	3
**O1'-$Na_{1/2}RuO_3$**	Hexagonal	$P\bar{3}:1m$	a = 5.1876(9) Å<br>c = 4.906(1) Å	1
