# Supplementary material for: Coulombic self-ordering upon charging a large-capacity layered cathode material for rechargeable batteries
Source: Nat Commun. 2019 May 16;10:2185. doi: 10.1038/s41467-019-09409-1 (PMC6522540; doi:10.1038/s41467-019-09409-1)
Supplement: Supplementary file 2 — Peer Review File [file 41467_2019_9409_MOESM2_ESM.pdf]

Editorial Note: This manuscript has been previously reviewed at another journal that is not operating a transparent peer review scheme. This document only contains reviewer comments and rebuttal letters for versions considered at Nature Communications.
